# Flexible, Water-Resistant and Air-Stable LiBH_4_ Nanoparticles Loaded Melamine Foam With Improved Dehydrogenation

**DOI:** 10.3389/fchem.2020.00045

**Published:** 2020-02-04

**Authors:** Yanping Fan, Dandan Chen, Zhenluo Yuan, Qiang Chen, Guangxin Fan, Dan Zhao, Baozhong Liu

**Affiliations:** ^1^College of Chemistry and Chemical Engineering, Henan Polytechnic University, Jiaozuo, China; ^2^School of Materials Science & Engineering, Henan Polytechnic University, Jiaozuo, China

**Keywords:** hydrogen storage materials, LiBH_4_, melamine foam, flexibility, air-stable

## Abstract

Flexible, water-resistant, and air-stable hydrogen storage material (named PMMA-LiBH_4_/GMF), consisting of LiBH_4_ nanoparticles confined by poly (methylmethacrylate) (PMMA) and reduced graphene oxide (rGO) modified melamine foam (GMF), were prepared by a facile method. PMMA-LiBH_4_/GMF can recover original shape after compression at the strain of 50% and exhibits highly hydrophobic property (water contact angle of 123°). Owing to the highly hydrophobic property and protection of PMMA, PMMA-LiBH_4_/GMF demonstrates outstanding water-resistance and air-stability. Significantly, the onset dehydrogenation temperature of PMMA-LiBH_4_/GMF at first step is reduced to 94°C, which is 149°C less than that of LiBH_4_/GMF, and the PMMA-LiBH_4_/GMF desorbs 2.9 wt% hydrogen within 25 min at 250°C, which is obviously more than the dehydrogenation amount of LiBH_4_/GMF under the same conditions. It's our belief that the flexible, water-resistant and air-stable PMMA-LiBH_4_/GMF with a simple preparation route will provide a new avenue to the research of hydrogen storage materials.

## Introduction

Hydrogen has been considered as a promising energy carrier owing to its higher power density, green and abundant resources (Nielsen et al., 2010; Liu et al., 2016). However, the safe and efficient hydrogen storage technique is one of the key technical obstacles to limit the commercial application of hydrogen-fueled vehicles (Schlapbach and Züttel, 2001). Compared to traditional high-pressure and liquid hydrogen storage systems (Zhang et al., 2014), the solid-state metal hydrides are prospective to realize future hydrogen storage goals due to their safety, compactness and efficiency (Sakintuna et al., 2007). Lithium borohydride (LiBH_4_) is well-known as one of the most promising metal complex hydrides, which has high gravimetric and volumetric hydrogen densities of 18.5 wt% H_2_ and 121 kg H_2_/m^3^, respectively (Zhang et al., 2017; Wu et al., 2019). Unfortunately, the practical applications of LiBH_4_ are restricted because of the challenging thermodynamics, slow kinetics and the sensitivity to water and oxygen. Moreover, the reversibility of LiBH_4_ is also limited by the harsh rehydrogenation conditions (high temperature up to 600°C and high pressure up to 35 MPa H_2_) (Zhao et al., 2014; Zang et al., 2018).

To overcome the drawbacks of LiBH_4_, several approaches, including doping catalysts, nanoconfinement and partial cation substitution, have been developed to lower dehydrogenation temperature, to increase the hydrogen desorption capacity and promote the rehydrogenation reaction under mild conditions (Zhou et al., 2012; Cai et al., 2013). Nanoconfinement is also a viable way to improve the dehydrogenation performance by increasing surface areas and decreasing the diffusion lengths (Huang et al., 2015). Therefore, many efforts have been applied to confine LiBH_4_ in carbon, SiO_2_ and metal-organic frameworks (MOFs) (Gross et al., 2008; Liu et al., 2010; Ngene et al., 2010; Sun et al., 2011; Vajo, 2011). LiBH_4_ confined in nanoporous carbon materials exhibits lower temperatures of phase transformation, melting, and dehydrogenation temperature as compared with bulk material, and reduced the formation of B_2_H_6_ (Gross et al., 2008; Liu et al., 2011). Cho et al. (2016) reported an environmentally stable hydrogen storage material of Mg nanocrystals encapsulated by atomically thin and gas-selective reduced graphene oxide (rGO) sheets, exhibits distinguishingly dense hydrogen storage (6.5 wt% and 0.105 Kg H_2_ per liter in the total composite) (Hecchetto et al., 2009). Gosalawit-Utke et al. (2014) reported that LiBH_4_ was confined by poly(methyl methacrylate)-co-butyl methacrylate (P(MMA-co-BMA)), and the onset dehydrogenation temperature was reduced to ~80°C. Zhao et al. (2010) reported a water-resistant system of ammonia borane (AB) confined by poly(methyl acrylate) (PMA), and found the dehydrogenation performance of AB was improved. An air-stable crystalline Mg/PMMA nanocomposites rapidly uptaked hydrogen (<30 min at 200°C) with high capacity (~6 wt% in Mg, ~4% overall) in the absence of heavy-metal catalysts (Jeon et al., 2011). PMMA, owning to the high permeability ratio of H_2_/O_2_, is better than other similar polymers in terms of gas selectivity. Therefore, PMMA can be used in hydrogen storage to keep away the water and oxygen but let the hydrogen get in or out freely (Wang et al., 2008; Liang et al., 2011). Huang et al. (2014) reported that LiBH_4_ nanoconfined in PMMA exhibits excellent hydrophobic ability and dehydrogenation. However, LiBH_4_/PMMA may be stiff and rigid due to the glass state of PMMA at room temperature, which may hinder the application of LiBH_4_/PMMA composites in the flexibility demanded fields.

In the present work, we reported a facile method to prepare flexible, water-resistant and air-stable PMMA-LiBH_4_/GMF for hydrogen storage and release, in which LiBH_4_ was confined by PMMA and GMF. The GMF was prepared by simple dipped coating and high temperature reduction. And then, we simply dipped GMF into LiBH_4_/PMMA solution (tetrahydrofuran, THF, as solvent), and dried the foam in the glove box under Ar atmosphere at room temperature for 72 h. Owing to the super-absorbent property, GMF can adsorb a large amount of THF solution as well as the solutes of LiBH_4_ and PMMA in the THF solution. After drying, PMMA-LiBH_4_/GMF, consisting of PMMA confined LiBH_4_ nanoparticles, can be successfully prepared. It is found the prepared PMMA-LiBH_4_/GMF is flexible, which can be compressive and maintains the elasticity for many times. Moreover, PMMA-LiBH_4_/GMF is also found to be highly hydrophobic, and the contact angle reached 123°, indicating there is water-resistant property for the new foam. The hydrophobic surface of the foam prevents water from permeating into the pores of the foam and PMMA shell on the LiBH_4_ nanoparticles, which also provides additional protection of LiBH_4_ nanoparticles to keep away from contacting with O_2_. Most importantly, the onset decomposing temperature of LiBH_4_ in the nanocomposite foam is reduced from 285 to 94°C and the main dehydrogenation temperature also decreased from 470 to 340–380°C. Unlike the common LiBH_4_ and LiBH_4_/PMMA nanocomposites, our PMMA-LiBH_4_/GMF exhibits flexibility, water-resistance, air-stability, and improved hydrogen releasing properties.

## Experimental

### Materials

Lithium borohydride solution (2.0 M LiBH_4_ in tetrahydrofuran) was purchased from Aladdin, PMMA was provided by TCI. All the reagents were used without any further purification and were stored and handled in a glove box (Etelux Lab 2000) equipped with an Ar recirculation system, and the water and hydrogen were kept below 0.1 ppm to prevent the oxidation.

### Preparation of PMMA-LiBH_4_/GMF

A two-step process was employed to prepare the PMMA-LiBH_4_/GMF materials. Firstly, GMF was prepared as the literature reported by Zhu et al. (2015). Briefly, melamine foam (MF) was cut into blocks and then ultrasonically cleaned using ethanol and deionized water, successively. The blocks were dried at room temperature. Then, the obtained clean MF was dipped into a graphene oxide (GO) suspension (5 mg/mL), and squeezed/released process in GO suspension was repeated for three times. Subsequently, GMF was obtained by heating the foam at a fixed temperature of 160°C for 6 h at an atmospheric environment to reduce GO into reduced graphene oxide (rGO). Secondly, PMMA-LiBH_4_/GMF was prepared by impregnating GMF into a PMMA-LiBH_4_ solution (tetrahydrofuran, THF, as solvent) with PMMA concentration of 70 mg/mL, then the modified foam was dried in the glove box at room temperature for 72 h. There was only an organic gas adsorption device in the glove box. An operation to change the atmosphere in glove box by Ar every 18 h to reduce the concentration of THF until the THF was completely dried.

### Characterizations

The microstructure was observed by scanning electron microscope (SEM, Zeiss Supa 50VP, Germany) with 15 kV. The phase structure of samples was characterized by X-ray diffraction (XRD) using a Smart-lab, CuKα radiation as a light source. All the XRD measurements were conducted at room temperature, and the scanning range of 5–70°. Contact angle was measured by contact angle meter (JC2000C, Shanghai). The compressibility of the foams was tested by the universal testing machine (WSM-10KN, Changchun).

### Hydrogen Storage Measurements

The dehydrogenation behavior was measured by thermogravimetry (TG, SDT, Q600) connected directly with quadrupole mass spectroscopy (MS, HPR 20, QIC). The samples were heated at a temperature range from room temperature to 500°C at a heating rate of 4°C/min with an argon purge rate of 90 mL/min. A Sievert's apparatus purchased from Zhejiang University was used to determine the temperature dependence of the hydrogen desorption and absorption behavior. For each test, ~0.1 g of the as-prepared samples were loaded into a stainless steel tube reactor within a glovebox, the reactor was then connected to the Sieverts-type apparatus. For the isothermal experiment, the sample was quickly heated to the desired temperature, and then the temperature was maintained during the following test.

## Results and Discussion

### Preparation of PMMA-LiBH_4_/GMF

A two steps process was used to prepare PMMA-LiBH_4_/GMF: (1) Graphene-coated melamine foam (GMF) was firstly prepared by dipping clean MF into a graphene oxide solution, and then the foam was heated to make the GO be reduced into reduced graphene oxide (rGO), (2) GMF was immersed into the LiBH_4_/PMMA THF solution, and then the foam was allowed to evaporate THF and form PMMA confined LiBH_4_ nanoparticles in the G-Melamine matrix.

**Figure 1:**
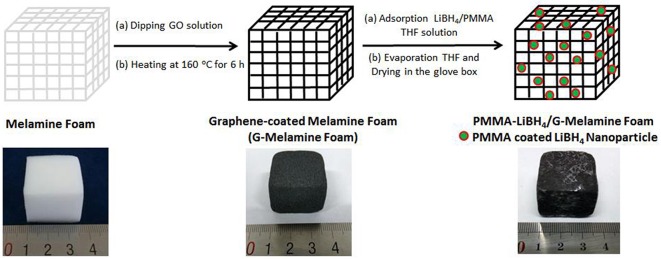
Illustration of preparation of PMMA-LiBH_4_/GMF.

As described above, PMMA-LiBH_4_/GMF was prepared by a facile two-step process. The detailed preparation route is shown in Figure 1. As shown in Figure 1, the MF shows the color change from white to black after thermal reduction of GO. The PMMA and LiBH_4_ remain in the GMF as the evaporation of THF solvent. Moreover, the size of LiBH_4_ nanoparticles can be controlled owing to the confinement effect of PMMA. In conclusion, PMMA-LiBH_4_/GMF can be facile prepared by the simple dipping-evaporation process.

### Morphology of PMMA-LiBH_4_/GMF

**Figure 2:**
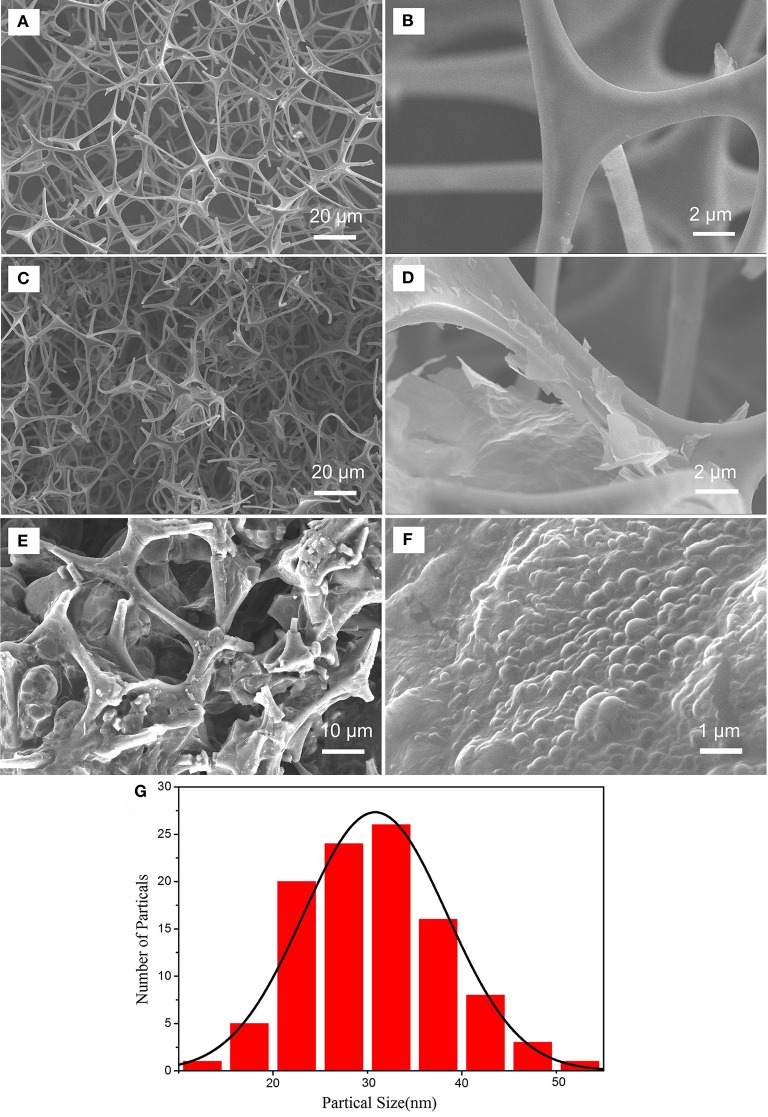
SEM images of MF **(A,B)**, GMF **(C,D)**, PMMA-LiBH_4_/GMF **(E,F)** and particle distribution of LiBH_4_ nanoparticles in PMMA-LiBH_4_/GMF **(G)**.

The morphologies of MF and GMF were observed by SEM as shown in Figures 2A–D. The SEM images of MF show that the pure MF is a 3D porous structure with pore diameters ranging from tens to hundreds of micrometers. Figure 2B exhibits the smooth sponge skeletons. After the introduction of graphene, the GMF keeps the 3D porous structure (Figure 2C), indicating that the network structures of MF are not damaged when the thermal reduction process of GO. However, the sponge skeletons of GMF are rougher than those of MF, this could be related to the presence of reduced graphene oxide (rGO) on the surface of sponge skeletons.

The magnified image further demonstrates that rGO with folding edge is stacked on the surface of sponge skeletons (Figure 2D). The results suggest that the GMF is successfully prepared via the thermal reduction of GO over sponge skeletons. Figures 2E,F show that PMMA-LiBH_4_ nanoparticles can fill into the pore of GMF. The particle distribution of PMMA-LiBH_4_ nanoparticles is shown in Figure 2G. The average particles size is about 30 nm. The size decrease of LiBH_4_ nanoparticles as wrapped by PMMA was also reported by Li et al. (2014).

### XRD Analysis of PMMA-LiBH_4_/GMF

**Figure 3:**
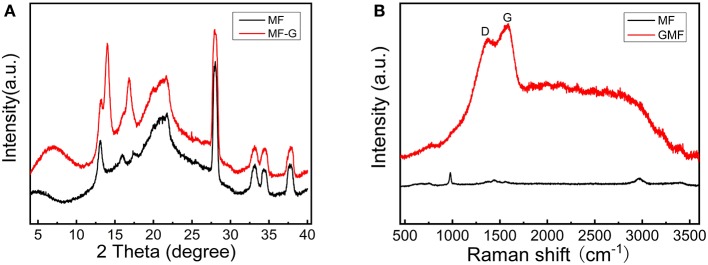
XRD patterns **(A)** and Raman spectra **(B)** of MF and GMF.

**Figure 4:**
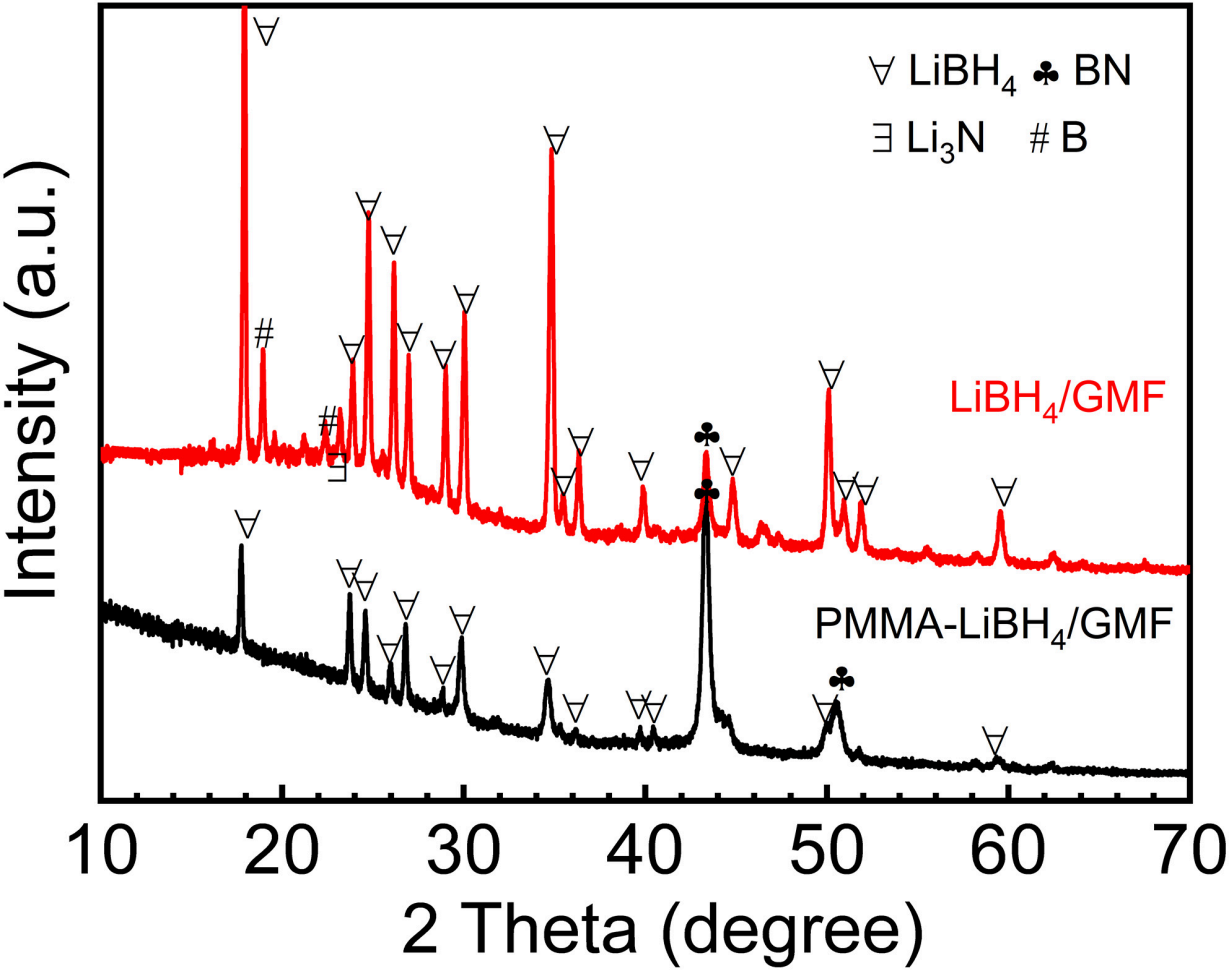
XRD patterns of LiBH_4_/GMF and PMMA-LiBH_4_/GMF.

The XRD technique was used to study the structure of different foams. As shown in Figure 3A, MF and GMF show similar XRD patterns, indicating the coating of graphene nanosheet do not affect the primary structure of MF. Moreover, the characteristic XRD peaks of layered graphene cannot be detected. However, the Raman spectra confirm the presence of graphene (Figure 3B). The Raman peaks attributed to the graphene can not be observed for MF, while the D and G graphene peaks of GMF associated with sp^2^ and sp^3^ bonds are observed in 1,370 and 1,580 cm^−1^, respectively. These results illustrate that GMF is obtained successfully in this experiment. As known from the literature (Zhao et al., 2010; Gosalawit-Utke et al., 2014), the crystalline of LiBH_4_ disappeared when LiBH_4_ was confined by PMMA, due to form amorphous and/or nanocrystalline structure for LiBH_4_. The XRD patterns of LiBH_4_/GMF and PMMA-LiBH_4_/GMF are shown in Figure 4. The XRD peaks corresponding to LiBH_4_ can be detected, and the LiBH_4_ phase is still dominant phase in both PMMA-LiBH_4_/GMF and LiBH_4_/GMF, illustrating that LiBH_4_ is successfully confined in PMMA and foam. However, the intensity of LiBH_4_ peaks in PMMA-iLiBH_4_/GMF is much lower than that of LiBH_4_/GMF, this can be related to the nano size that resulted by the confinement effect of PMMA. Meanwhile, for LiBH_4_/GMF, the peaks of BN, Li_3_N, and B can be detected, indicating LiBH_4_ and GMF reacted partially during the preparation. Besides LiBH_4_, only XRD peaks ascribed to the BN phase can be observed for PMMA-LiBH_4_/GMF, indicating the PMMA can effectively restrict the reaction between LiBH_4_ and GMF.

### Flexibility and Water-Resistance of PMMA-LiBH_4_/GMF

**Figure 5:**
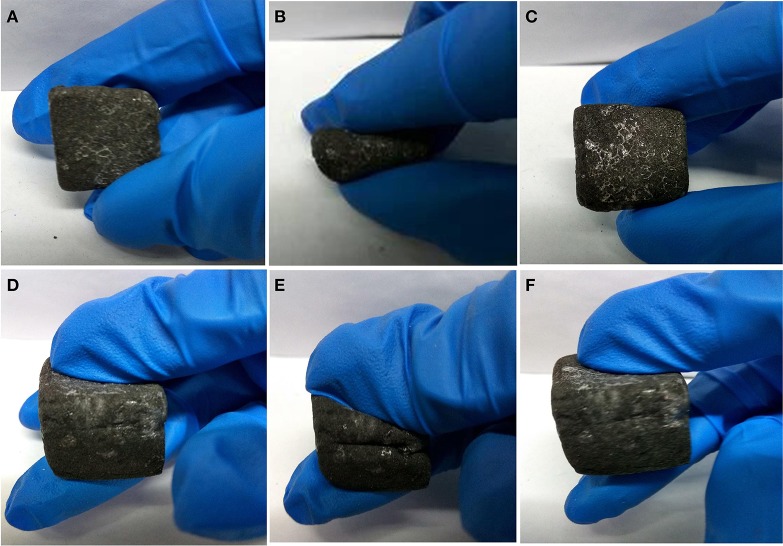
The flexibility and compressibility of PMMA-LiBH_4_/GMF: **(A–C)** and **(D–F)**.

In order to demonstrate the flexibility and compressibility of PMMA-LiBH_4_/GMF, the folding and compressive tests have been conducted as shown in Figure 5. After folding, the PMMA-LiBH_4_/GMF can completely recover to its original shape and size immediately (Figures 5A–C). Similarly, as the compressive force released, the foam also can rapidly recover to its original shape (Figures 5D,E). The results indicate our PMMA-LiBH_4_/GMF exhibits excellent flexibility though there is glassy PMMA on or in the foam.

**Figure 6:**
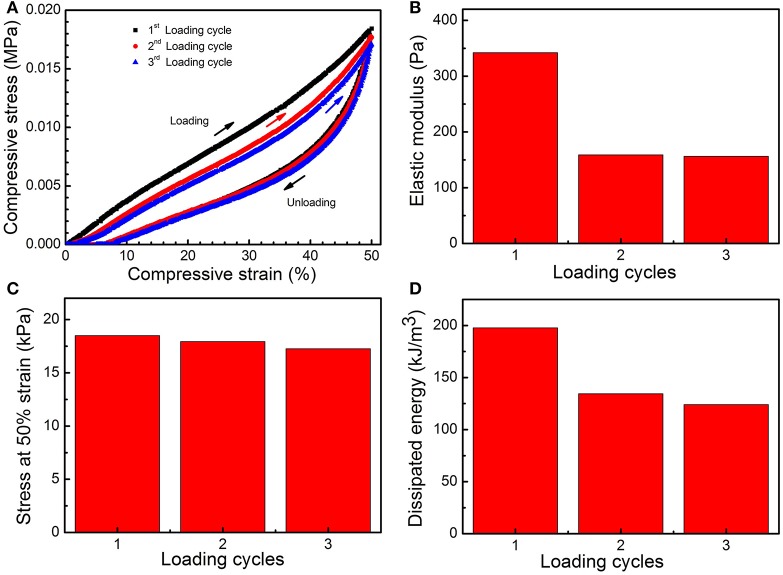
Hysteresis loops **(A)**, elastic modulus (E) **(B)**, stress at 50% strain **(C)**, and dissipated energies **(D)** of PMMA-LiBH_4_/GMF for the first, second, and third compression.

To further evaluate the flexibility of the PMMA-LiBH_4_/GMF, three compressive loading-unloading cycle tests were measured. As shown in Figure 6A, the foam shows obvious hysteresis loops for three compressive loading cycles. It is found the elastic modulus (E), the stress at 50% strain and the dissipated energies are 344/159/157 Pa, 19/18/17 kPa, and 200/134/123 kJ/m^3^ for the first, second, and third compression, respectively (Figures 6B–D). Herein, the mechanical properties of PMMA-LiBH_4_/GMF decrease after the first loading. However, the loading-unloading loops of second and third compression are almost overlapped, indicating the mechanical properties of the foam become stable after the first loading.

**Figure 7:**
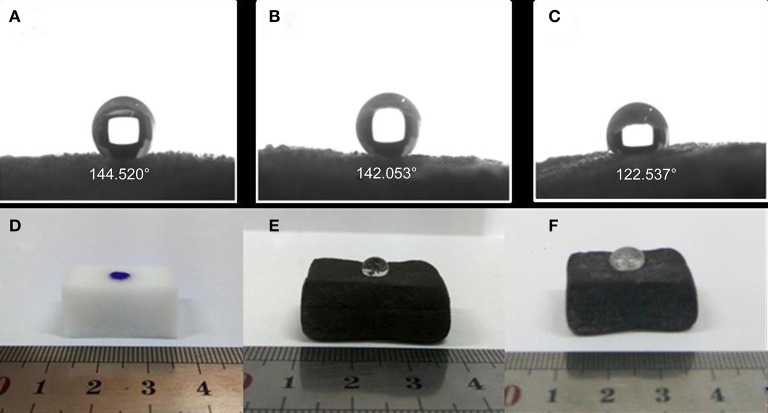
The contact angle of **(A)** GMF, **(B)** PMMA/GMF and **(C)** PMMA-LiBH_4_/GMF and the picture of water droplet (stain with methylene blue) on the surface of **(D)** MF, **(E)** GMF, and **(F)** PMMA-LiBH_4_/GMF.

It's well-known that graphene modified MF is superhydrophobic, and it's expected our PMMA-LiBH_4_/GMF will be also highly hydrophobic to resist water diffused into the foam, and subsequently reacted with LiBH_4_ nanoparticles. In order to determine the hydrophobicity of PMMA-LiBH_4_/GMF, the contact angle was measured. As shown in Figure 7, the water contact angle of GMF, PMMA/GMF and PMMA-LiBH_4_/GMF is 145, 142, 123°, respectively. As reported in literature (Li et al., 2014), GMF exhibited the best hydrophobicity. Although the contact angle slightly decreased, GMF still possessed high hydrophobicity after adding PMMA. Most interestingly, our PMMA-LiBH_4_/GMF also preserves high hydrophobicity of 123° (Figure 7C). For more visible, a water droplet is dropped on the surface of various foams. Consistently, water droplet was quickly permeated into the original MF, whereas water droplet on the GMF and PMMA-LiBH_4_/GMF shows semicircle shape. Therefore, our PMMA-LiBH_4_/GMF is highly hydrophobic, which will protect the LiBH_4_ nanoparticles away from the contact with moisture in the air.

### Dehydrogenation Properties of PMMA-LiBH_4_/GMF

**Figure 8:**
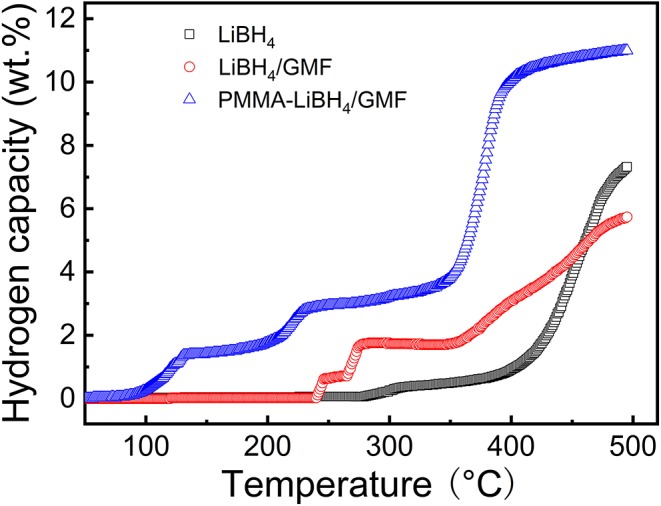
Temperature programmed dehydrogenation (TPD) curves of pure LiBH_4_, LiBH_4_/GMF, and PMMA-LiBH_4_/GMF.

Figure 8 shows the TPD curves of PMMA-LiBH_4_/GMF. It is obvious that the dehydrogenation temperature of LiBH_4_ is significantly reduced by combining with PMMA and GMF. Pure LiBH_4_ starts to decompose at 290°C and rapidly release hydrogen at around 400°C. The dehydrogenation of LiBH_4_ can be enhanced by the introduction of GFM. For LiBH_4_/GMF composite, the onset dehydrogenation temperature is reduced to 243°C, which is 47°C lower than that of pure LiBH_4_, moreover, three dehydrogenation stages can be observed, and the total dehydrogenation is inferior to the pure LiBH_4_ under the studied temperature region. More interestingly, the majority of dehydrogenation temperature significantly shifts to lower temperature after the introduction of PMMA. The onset dehydrogenation temperature of PMMA-LiBH_4_/GMF at first step is reduced to 94°C, which is 149°C less than that of LiBH_4_/GMF, and the second step is also lowered by 56°C, from 265 to 209°C. Moreover, the dehydrogenation amounts of PMMA-LiBH_4_/GMF at the three steps are more than that of LiBH_4_/GMF. More importantly, about 7 wt% hydrogen can be fast released at the temperature range from 350 to 400°C. The total dehydrogenation amounts of PMMA-LiBH_4_/GMF are about 11 wt%, which are obviously higher than those of pure LiBH_4_ and LiBH_4_/GMF when the temperature was lower than 500°C. Therefore, the co-existence of GMF and PMMA can greatly improve dehydrogenation capacity of LiBH_4_.

**Figure 9:**
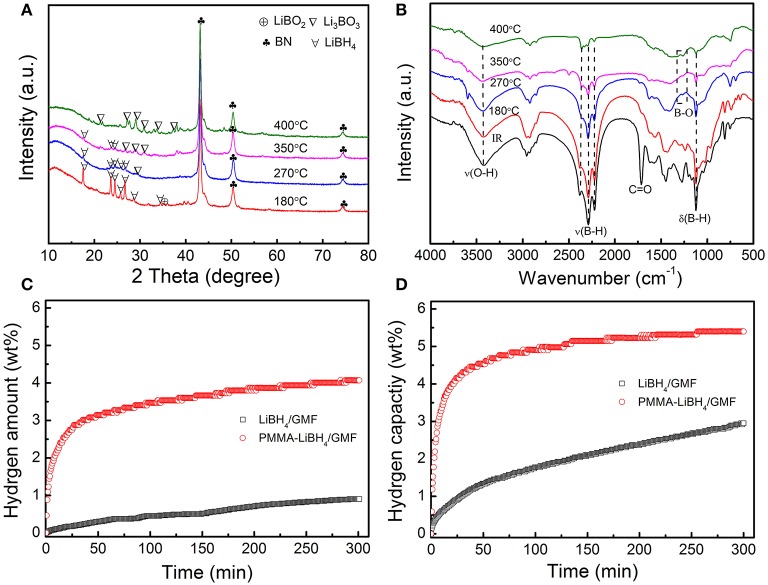
XRD **(A)** and FTIR **(B)** patterns of dehydrogenated of PMMA-LiBH_4_/GMF at 180, 270, 350, and 400°C and the isothermal hydrogen desorption curve of LiBH_4_/GMF, PMMA-LiBH_4_/GMF **(C,D)**.

To understand the underlying reason for the excellent dehydrogenation performances of PMMA-LiBH_4_/GMF, XRD patterns and FTIR spectra of PMMA-LiBH_4_/GMF at different dehydrogenation states during hydrogen desorption process were conducted as shown in Figures 9A,B. Seen from the XRD in Figure 4, there is a partial reaction between LiBH_4_ and GMF during the preparation of PMMA-LiBH_4_/GMF. After dehydrogenation at 180°C, the XRD peaks at 2θ = 43.1, 50.4, 74.4° can be detected and was assigned to BN phase besides the peaks of LiBH_4_ (Figure 9A), which maintains a similar structure with fresh PMMA-LiBH_4_/GMF. In the FTIR spectra, B-H bond has a relatively weak intensity comparing with the fresh PMMA-LiBH_4_/GMF, which may be ascribed to the rise in temperature stimulating the reaction between LiBH_4_ and GMF. And synchronously no B-O bond is detected at 180°C (Figure 9B). Thus, the possible mechanism before 180°C conjectured is that GMF as a N source has a chemical reaction with LiBH_4_. When the dehydrogenation temperature increase to 270°C, the new XRD peak at about 30° appears, indicating Li_3_BO_3_ is formed. Here, LiBH_4_, BN are still the main components. The B-O bond is observed in the FTIR of the sample dehydrogenated at 270°C (Figure 9B). Based on these results, we come to the conclusion that the formation of Li_3_BO_3_ may be due to the occurred chemical reaction between LiBH_4_ and PMMA (O source) in the second dehydrogenation step. The dehydrogenation amount is relatively poorer under this temperature phase, which illustrates not too strong chemical reaction between PMMA and LiBH_4_ with increasing the temperature to 400°C, Li_3_BO_3_ and BN become the main peaks in XRD pattern of PMMA-LiBH_4_/GMF, and no LiBH_4_ can be detected. Additionally, seen from the FTIR spectra, B–O bond is existed, and the B–H bond gradually weakens and almost can not be resolved at 400°C. This phase presented an optimal dehydrogenation amount. This phenomenon attests that a strong chemical reaction occurs between PMMA and LiBH_4_. Based on XRD and FTIR results, the hydrogen release of PMMA-LiBH_4_/GMF foam mainly depends on the reaction between PMMA and LiBH_4_. Potentially, due to the formation of Li_3_BO_3_, the new reaction pathway of LiBH_4_ lead to the low dehydrogenation temperature and fast decomposition kinetics of PMMA-LiBH_4_/GMF. Additionally, the possible electrostatic interaction between B and O atom weakens the B-H bonds and lowers the hydrogen desorption temperature. Summarily, the existence of PMMA and GMF has an accelerated effect toward the dehydrogenation property of LiBH_4_.

In order to further study the effects of GMF and PMMA on the dehydrogenation properties of LiBH_4_, the isothermal hydrogen desorption experiments were also conducted (Figures 9C,D). PMMA-LiBH_4_/GMF desorbs 2.9 wt% hydrogen within 25 min at 250°C, which is obviously more than the dehydrogenation amount of LiBH_4_/GMF at the same conditions. Moreover, 4.2% hydrogen can be released from PMMA-LiBH_4_/GMF within 25 min at 350°C. Our PMMA-LiBH_4_/GMF exhibits excellent dehydrogenation performance, which is better than LiBH_4_/GMF and pure LiBH_4_ (Zhu et al., 2015). Combining the above experimental results, the initial onset dehydrogenation temperature values of 290, 243, and 94°C obtained from TPD curves can be ascribed to LiBH_4_, LiBH_4_/GMF, and PMMA-LiBH_4_/GMF, respectively. When compared with the initial onset dehydrogenation temperature of these materials, PMMA-LiBH_4_/GMF presents an excellent dehydrogenation property in LiBH_4_. And coincidentally, PMMA-LiBH_4_/GMF reveals an optimal total dehydrogenation capacity under uniform reaction system.

## Conclusion

In the present work, we provided a convenient route to synthesize flexible, water-resistant and air-stable PMMA-LiBH_4_/GMF with improved hydrogen releasing properties. GMF and PMMA can not only protect LiBH_4_ from water and oxygen, but also hinder growth and agglomeration of the particles Significantly, our PMMA-LiBH_4_/GMF can maintain the original flexible characteristics and hydrophobicity of GMF. More importantly, our PMMA-LiBH_4_/GMF exhibits excellent hydrogen desorption property, which is better than those of LiBH_4_/GMF and pure LiBH_4_. The improvement of dehydrogenation property of PMMA-LiBH_4_/GMF may be due to chemical effect between PMMA (or GMF) and LiBH_4_. We hope our PMMA-LiBH_4_/GMF provides a new avenue to the next generation hydrogen storage materials with novel functionalities.

## Data Availability Statement

All datasets generated for this study are included in the article/supplementary material.

## Author Contributions

YF and DC conducted the synthesis experiment, performance test, and paper writing. ZY participated in performance testing. GF, DZ, and QC participated in analysis of the results. QC and BL conducted the design, guidance, analysis of experiment results, and paper revision.

### Conflict of Interest

The authors declare that the research was conducted in the absence of any commercial or financial relationships that could be construed as a potential conflict of interest.
